# Comprehensive Characterization of Molecular Interactions Based on Nanomechanics

**DOI:** 10.1371/journal.pone.0003610

**Published:** 2008-11-03

**Authors:** Murali Krishna Ghatkesar, Hans-Peter Lang, Christoph Gerber, Martin Hegner, Thomas Braun

**Affiliations:** 1 National Center of Competence for Research in Nanoscience, Institute of Physics, University of Basel, Basel, Switzerland; 2 CRANN, SFI Nanoscience Institute, Trinity College, University of Dublin, Dublin, Ireland; 3 California Institute of Technology, Pasadena, California, United States of America; Massachusetts Institute of Technology, United States of America

## Abstract

Molecular interaction is a key concept in our understanding of the biological mechanisms of life. Two physical properties change when one molecular partner binds to another. Firstly, the masses combine and secondly, the structure of at least one binding partner is altered, mechanically transducing the binding into subsequent biological reactions. Here we present a nanomechanical micro-array technique for bio-medical research, which not only monitors the binding of effector molecules to their target but also the subsequent effect on a biological system *in vitro*. This label-free and real-time method directly and simultaneously tracks mass and nanomechanical changes at the sensor interface using micro-cantilever technology. To prove the concept we measured lipid vesicle (∼748*10^6^ Da) adsorption on the sensor interface followed by subsequent binding of the bee venom peptide melittin (2840 Da) to the vesicles. The results show the high dynamic range of the instrument and that measuring the mass and structural changes simultaneously allow a comprehensive discussion of molecular interactions.

## Introduction

**Figure 1:**
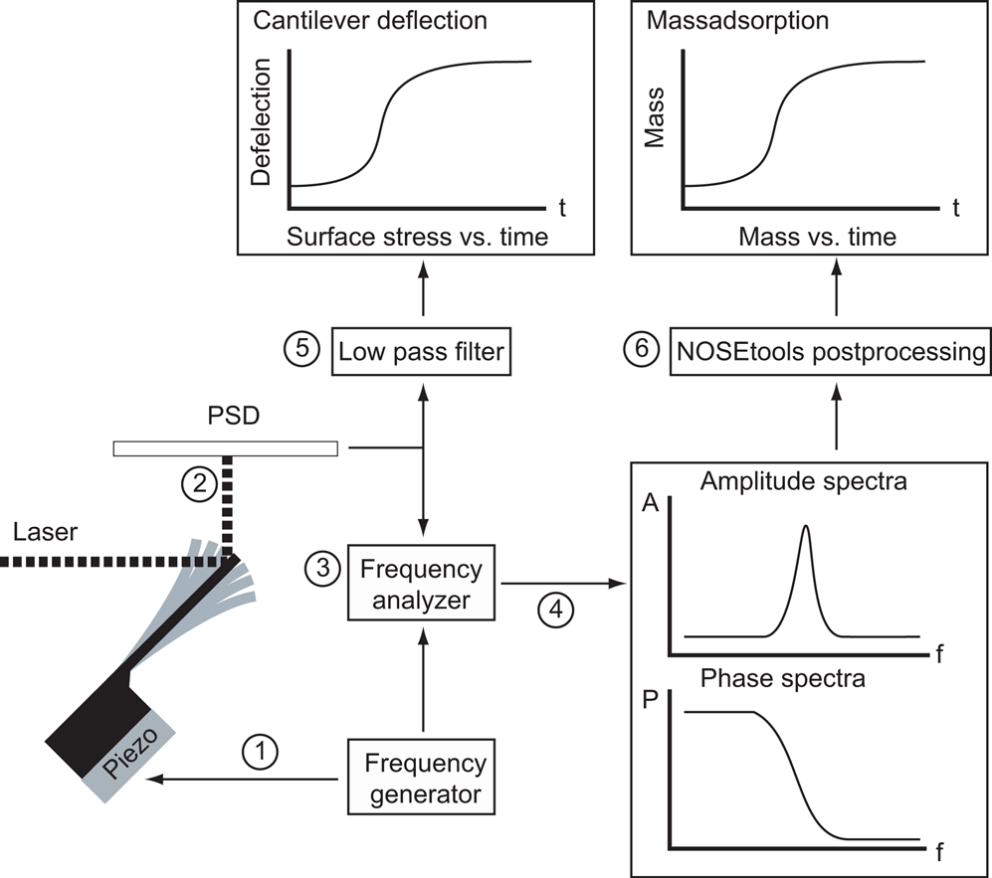
Schematic of the homemade measurement set-up for combined mode measurements. For more information see also Braun *et al.* 2007 [15]. An array of silicon cantilevers was mounted onto a piezo element. A sinusoidal excitation signal generated from a network analyzer swept the requested frequency range vibrating the cantilevers (1). The laser beam deflection detection technique was used to monitor the response of individual cantilevers (2). A frequency analyzer (3) compared input- and output signals and continuously recorded amplitude and phase spectra (4) as well as static deflection (bending) of the cantilevers (5). NOSETools software was used to analyze the spectra and extract the mass adsorption on the cantilever (postprocessing, 6). [5], [15], [21], [35] A scanning electron microscopy image of a cantilever array is shown in the supplemental Figure S1.

This work focuses on the development and testing of an instrument that measures the integral nanomechanics of molecular interactions [1]–[3]. This device relies upon the unique ability of thin cantilevers [4] to detect both the mass of the adsorbed molecules and nanomechanical changes on the cantilever interface, e. g. structural rearrangements. The mass is measured *via* the resonance frequency of the cantilever (dynamic mode) [5], [6]. Structural changes are detected by static bending of cantilevers (static mode) as demonstrated recently [7]–[9]. Here nanomechanical interaction changes generate a surface-stress difference between the asymmetrically functionalized cantilever interfaces forcing the beam to bend [10]–[14]. Technical details of the method are presented in Figure 1. In summary, the micro-fabricated cantilever arrays (supplemental Figure S1) are actuated for a given frequency range and the response is recorded as amplitude and phase spectra for the individual cantilever sensors. These spectra are post-processed and various physical properties of the system can be extracted, such as the adsorbed mass (dynamic mode) and the static cantilever bending (static mode) [15].

To validate the concept we measured molecular interactions between synthetic melittin and lipid vesicles. Melittin is the main component of the bee venom from the European honeybee and is responsible among other constituents for the hemolytic activity of this poison [16]. The small peptide (2.84 kDa) and its interactions with lipid membranes were studied in detail using a combination of various biophysical methods [16]. The peptide binds spontaneously to lipid membranes, forms an α-helix, inserts into the membrane, aggregates and creates channels [17]. While the exact pore formation mechanism is still uncertain it is known that the binding and channel-formation of melittin into vesicle bilayers involves nanomechanical changes. The vesicle mass increases and as visualized recently the insertion of the peptide leads to mechanical forces that subsequently push circumfluent membranes [9], [18].

## Results

**Figure 2:**
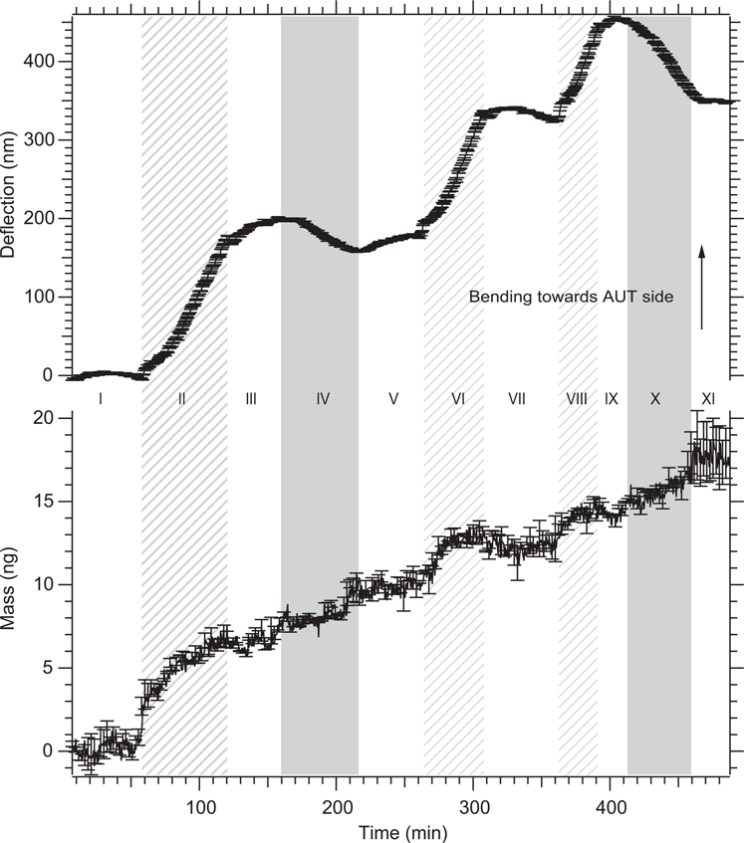
Combined mode measurements of vesicle and melittin adsorption on the cantilever sensor. The positive controls were pre-functionalized in such a way that vesicles only bind to the upper cantilever surface and melittin does not bind at all. The lower graph displays the mass adsorption and the upper graph reveals the surface stress development measured simultaneously. Note that the surface stress represents the differential signal between the positively and negatively functionalized cantilevers (two cantilevers each). The experiment was performed in 11 sections: (I) Baseline recording in buffer. (II, VI, VIII) Injections of 500 ng/ml DOPC vesicles. (III, V, VII, IX, XI) Buffer injections. (IV, X) Melittin injections (1 µM). Note that during the injection of melittin, the adsorbed mass is initially underestimated due to the high friction of the protein-solution, see also Braun *et al.*, 2005 [5] for a discussion. During the subsequent buffer injection the correct mass is measured.

Supplemental Figure S2 (panel A) depicts the workflow of the main experiments and the results are shown in Figure 2. The adsorption of lipid to the cantilever has to be controlled carefully since asymmetrical functionalization of the cantilevers was crucial for detection of the static cantilever bending (a single sided coating was not a prerequisite for the mass adsorption signal). This was achieved by a specific pre-functionalization of the sensor interfaces.

### Functionalization of cantilevers

First, the upper side of the cantilever array was coated with a 20 nm gold layer onto a 3 nm Titanium adhesion layer. For the positive controls, the cantilevers were pre-functionalized by a self-assembled monolayer (SAM) of 11-Aminoundecan-1-thiol (AUT) on the gold-coated cantilevers using liquid-filled glass capillaries [19]. This resulted in a positive charge selectively formed on the upper cantilever surface. The negative controls remained untreated. After the SAM formation, the complete array was immersed in casein to block “unspecific” binding of melittin and lipid to the silicon [20]. A series of mass-adsorption control experiments were conducted to carefully direct the specific binding or blocking of lipid-vesicles. Double sided AUT-functionalized cantilevers bound approximately double the amount of lipids than single sided functionalized ones as shown in supplementary data S1. Therefore we conclude that the cantilevers pre-functionalized with an AUT SAM on the gold-coated cantilever promote specific binding of lipid vesicles. We also found that casein blocks efficiently the binding of lipid-vesicles and Melittin to silicon and gold (see supplementary data S1 and S2). Note that this treatment of the cantilever not only promotes the specific binding of lipid vesicles to AUT pre-functionalized interfaces but also blocks by electrical repulsion the direct adsorption of the melittin peptide without preceding lipid-vesicles immobilization.

### Binding experiments

**Figure 3:**
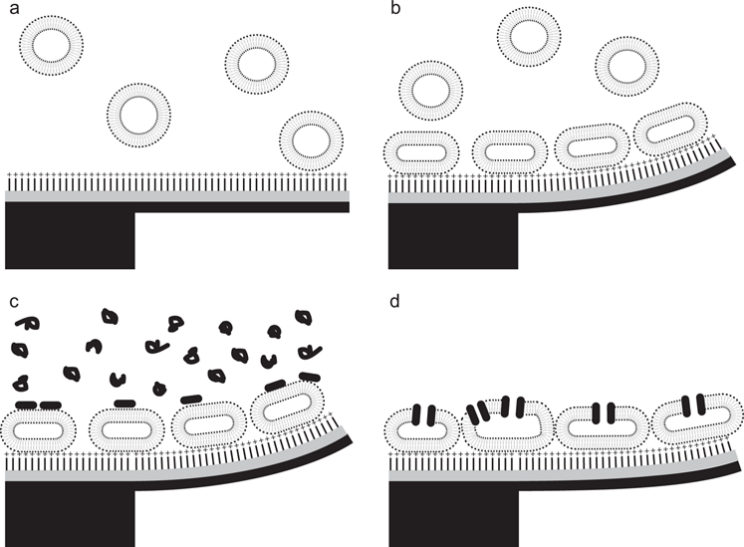
Molecular model of the nano-mechanical changes on the sensor interface explaining the data. Without vesicles the pre-functionalized cantilever (AUT SAM on gold) is straight (a). Adsorption of vesicles on the cantilever surface bends the cantilever upwards driven by the interaction forces between the cantilever and the vesicles, which are flattened by this interaction (b). During the peptide injection, the melittin molecules first bind to the vesicle surface (c), and later insert into the membrane and form channels by oligomerization (d).

Figure 2 shows the simultaneously measured mass adsorption and surface stress for three vesicles (500 ng/ml lipid) and two melittin (1 µM) solution injections (sections I to XI). The differential signal is shown obtained by the subtraction of the average of the negative controls from the average of the positive controls. Table 1 lists the mass and deflection *changes* during injection of lipid or melittin solutions. We used lipid and buffer conditions known to procure the membrane insertion and channel formation as reported previously [17]. After recording a baseline (section I), vesicles were injected with a concentration of 500 ng/ml (Fig. 3, section II). A mass increase of 6.4±0.06 ng (standard error, Table 1) is observed. The surface stress difference between the AUT functionalized top-side and the casein passivated silicon bottom side of the cantilever leads to an upward bending (towards the AUT) of the cantilever by 185±1.2 nm during vesicle adsorption. After vesicle injection buffer was flushed through the measurement chamber again (section III) before melittin (1 µM) was injected once (section IV) resulting in a mass increase of around 3.3±0.06 ng. Simultaneously the cantilever bent down by 14.6±1.1 nm. These surface stress changes are in close agreement with previously reported static mode measurements [9]. The injection sequence was complemented with two additional vesicle exposures (sections VI and VIII) and a final melittin dose (X) exhibiting the same qualitative mass and deflection changes. Every vesicle and melittin injection was terminated by a buffer wash. This procedure removes not only weakly bound molecules but also ensures that the signal is not due to the liquid rheology [21]. A summary of the mass and deflection changes is given in Table 1 and more details are available in the supplemental Table S1.

**Table 1:** Mass and deflection changes measured simultaneously.

Effector	Section	Mass (ng)	Deflection (nm)
Vesicles	II	6.35±0.006	185±1.1
Melittin	IV	3.34±0.006	−14.6±1.6
Vesicles	VI	2.37±0.006	162±1.69
Vesicles	VIII	1.8±0.008	121±1.69
Melittin	X	5.27±0.15	−105±1.69

The section numbers correspond the labeling of figure 2. Errors are given as standard errors. More detailed numbers about the statistics are presented in the supplementary table S1.

## Discussion

The results in Figure 2 demonstrate the high dynamic range of this method measuring large and small masses in a reproducible manner over several injections in the same experimental series. Furthermore, these experiments show that mass adsorption as well as static bending of the cantilever can be recorded simultaneously, as previously demonstrated for non-biological gas measurements [22] and in liquids studying temperature changes [15]. We interpret the mass changes during the injection of vesicles as lipids binding to the AUT functionalized side of the cantilevers. During the injection of melittin this mass change is attributed to the binding of the small peptide to the lipid vesicles. The change in deflection of the cantilever is construed as a result of the interaction through electrostatic forces between lipid-vesicles and the cantilever [23]. During the melittin injection we interpret the change in deflection as melittin binding and insertion into the lipid bilayer thus forming channels. A schematic of the (simplified) molecular interpretation is shown in Figure 3. The qualitative results are in excellent agreement with current models of the binding and melittin action on and in lipid bilayers [16], [17], [24]. Interestingly, the mass changes follow exactly the injection of the adsorbents (vesicles, melittin) whereas deflection alterations are also observed during the injections of buffer. Such changes we interpret as global structural rearrangements taking place on the cantilever surface after vesicle or melittin binding respectively. All our data show that the lipid binds in the form of a vesicle layer on the AUT functionalized side of the cantilever (see also supplementary data S1 and S2). From the mass-adsorption we can determine a molecular protein to lipid ratio of 1∶10 mol/mol (0.5w/w) for the first melittin injection. This is in the range of typical melittin to lipid ratios and the bilayer structure is reported to stay intact for this particlar mixture [25]. For the second melittin injection (Fig. 2, section X), a protein to lipid ratio of 1∶3 mol/mol (0.8w/w) was measured (taking the complete adsorbed lipid and protein mass into account, see also supplemental tables S1). This ratio is reported to destabilize the lipid structure [25]. Indeed, the static mode signal does not exhibit any change in deflection after the last melittin injection (section XI). This is in contrast to section V following the first melittin incubation (section IV). Here the cantilever shows an upward bending similar to the signal during vesicle adsorption but no mass changes occurs. This fact indicates that the observed deflection changes are due to structural rearrangements and not caused by electrostatic repulsion between the melittin peptides (see also supplementary data S3). The bending of the cantilever does not correlate linearly with the amount of the bound melittin. For the first melittin injection (section IV) the relative deflection change is −4.7 nm/ng and for the second injection it is −20 nm/ng. This is expected for cooperative processes with many interaction sites involved. Following the first melittin injection a net increase in vesicle mass was observed with subsequent lipid injections (sections VI and VIII). This is explained by the property of melittin to disturb lipid bilayers leading to association/fusion of lipid vesicles as reported previously [25], [26].

A more quantitative discussion of the surface stress development observed in Figure 2 includes the fact that static mode measurements do not only depend on the amount of absorbed (melittin) molecules, but also the nature of the adsorbents and their specific molecular interactions. Note that surface stress is an intensive dimension (in contrast to the measured mass) and is the result of the ensemble of interactions between the molecules adsorbed on the sensor surface. In our case, the vesicular structure of the lipid on the cantilever interface complicates the geometrical arrangement of the adsorbed melittin peptides. Only in-plane force components in the direction of the cantilever main-axis are contributing to the measured surface stress [13]. Therefore the average mechanical lipid-expansion work of a melittin peptide contributing to the cantilever bending is significantly underestimated and requires extensive corrections (not presented here).

Differently designed melittin binding experiments (supplementary figure S2, panel B), confirming the results discussed above, are presented in Supplementary data S3. For the negative control reference, every second cantilever was pre-incubated with melittin. Interestingly, the mass measurement reveals that melittin bound to the positive as well to the negative control during the *in situ* binding experiments. However, the pre-incubated cantilevers did not exhibit any significant change in deflection (surface stress). This finding demonstrates that electrostatic interactions are not dominating the forces that lead to cantilever bending but rather an nanomechanical expansion of the lipid layer as reported before [9].

For the combined mode (static and dynamic recording) we experienced that the optimized thickness of the cantilever is about 1 µm. The sensitivity for static mode increases with lower spring constant but the sensitivity of the dynamic mode increases with higher frequencies (higher spring constants). In our experiments presented here we used cantilever-arrays with soft spring constants (0.02 N/m) but measured at higher modes (mode 13, 14 or 15) of vibration to increase the sensitivity [5], [27], [28]. Using supported amphiphilic polymer [29] or lipid (bi-) layers [30] would even enhance functionalization efficiency and static mode information content. This would allow a more precise and quantitative interpretation of the static mode information. This technique has the potential to replace Langmuir monolayer assays [31] with the advantages that in addition to the surface stress signal the number of adsorbed molecules could also be measured.

This work demonstrates for the first time that simultaneous and *direct* measurement of nanomechanical (structural) changes and mass adsorption can be performed on the same sensor platform in a liquid environment. Other techniques based on optical detection in combination with Quartz crystal mass balance (QCM) techniques [32] were successfully applied for vesicle adsorption measurements but the different signals were recorded independently.

### Conclusions and outlook

Our results show firstly that this sensor can measure large ultrastructures and small peptides successively and secondly that the combined measurement of two intrinsic physical properties allows a comprehensive description of molecular interactions. In summary, the dynamic mode mass measurements provide binding information, which does not depend on the nature of the detected system, whereas the static mode provides information about the characteristics of the interactions system, e.g. global structural changes as demonstrated here. We strongly believe that the combined measurement will be established as a general tool to characterize molecular interactions. Systems biology [33] needs tools that not only detect binding partners, but also provide further information on structural changes to comprehend higher organizational levels. Cantilever sensors are perfectly suited for this purpose because the nano-mechanical measurement principle monitors both the binding of effector molecules to their partner and also the subsequent effect on a biological system *in vitro*. This sensor characteristic is unique and allows intriguing applications in nano-medicine as a new method for drug screening and diagnostics.

## Materials and Methods

### Materials

Dioleylphosphatodycholine (DOPC) was purchased from Avanti Polar lipids Inc, USA; 11-Aminoundecanthiol (AUT), other chemicals such as melittin and water (HPLC grade) from Sigma-Aldrich, Switzerland. Throughout all experiments the same buffer at pH 7.4 (10 mM HEPES 107 mM NaCl and 1 mM Na_2_EDTA) was used. Silicon cantilever arrays were obtained from the IBM research laboratories, Zurich, Switzerland.

### Cantilever preparation

Arrays with eight cantilevers were cleaned in piranha solution (H_2_SO_4_ (96%)∶H_2_O_2_(31%) = 1∶1) followed by a wash step in water. After repeating the first cleaning step, a final cleaning in NH_3_ (30%) ∶ H_2_O_2_ (31%) ∶ water = 1∶1∶1 for 20 min was performed complemented with a final washing step in water (2×10 min). Finally, the cantilever arrays were incubated for 5 min in 2-propanol and dried. A 20 nm gold layer was deposited (rate: 3 nm/min) on the freshly cleaned silicon with a 3 nm (3 nm/min) titanium adhesion-layer in between using a Balzers MED 010 (Balzers, Liechtenstein) thermal evaporation apparatus. The differential functionalization between negative and positive control was accomplished using a capillary device as described elsewhere[34]. Every second cantilever (positive control) was incubated in an ethanol solution of AUT (1 mM) for 1 h. The formed SAM provides a net positive charge on the cantilever in buffer solution. Finally, unspecific binding sites were blocked by incubating the complete cantilever array in a 1 mg/ml casein solution for 10 min. The casein bath was always prepared fresh by shaking the protein sulution for at least 2 h at 37°C. At the end, the solution was filtered (0.2 µm pore size).

### Vesicle preparation

Unilamelar DOPC vesicle solutions were produced as described elsewhere.[24] In short, the chloroform-dissolved lipid (DOPC) were first dried under Argon and then kept under vacuum over night. The lipid films were hydrated in buffer (final concentration of 10 mg/ml) under heavy vortexing. Six freeze/thaw cycles were performed followed by extruding at a concentration of 5 mg/ml through a 100 nm filter pore (Whatman, UK). Dynamic light scattering (ALV-Langen) of the vesicles revealed a hydrodynamic radius of 100 nm.

### Melittin

The synthetic melittin was dissolved in buffer at a concentration of 88 µM as determined by light adsorption measurement at 280 nm using a coefficient of 5570 M^−1^ cm^−1^. The solved peptide was stored at −20°C prior to further use and diluted to 1 µM just before the experiment.

### Binding experiments

The pre-functionalized cantilever was mounted in the measurement chamber (Volume 6 µl) without drying. Different solutions (see Fig. 2) were injected at a flow-rate of 10 µl/min. In the first phase (section I Fig. 2) a baseline was recorded and this data was used for calibrating of the virtual mass as described in detail elsewhere [5].

### Data analysis

All data processing was performed using the NOSEtools software (information and download at http://web.mac.com/brunobraun/iWeb/NOSETools/) written in the IGOR Pro programming environment (www.wave metrics.com). The signals of individual cantilevers with identical functionalization were averaged after alignment as described elsewhere. [35] Details of the measurement method and digital data processing are described in Braun et al., 2007 [15].

## Supporting Information

Figure S1 (0.22 MB PDF)

Figure S2 (0.84 MB DOC)

Data S1 (0.33 MB PDF)

Data S2 (0.37 MB PDF)

Data S3 (0.20 MB DOC)

Table S1 (0.07 MB DOC)
