# Supplementary material for: Comprehensive Characterization of Molecular Interactions Based on Nanomechanics
Source: PLoS One. 2008 Nov 3;3(11):e3610. doi: 10.1371/journal.pone.0003610 (PMC2572191; doi:10.1371/journal.pone.0003610)
Supplement: Figure S1 — (0.22 MB PDF) [file pone.0003610.s001.pdf]

Supplementary Figure S1

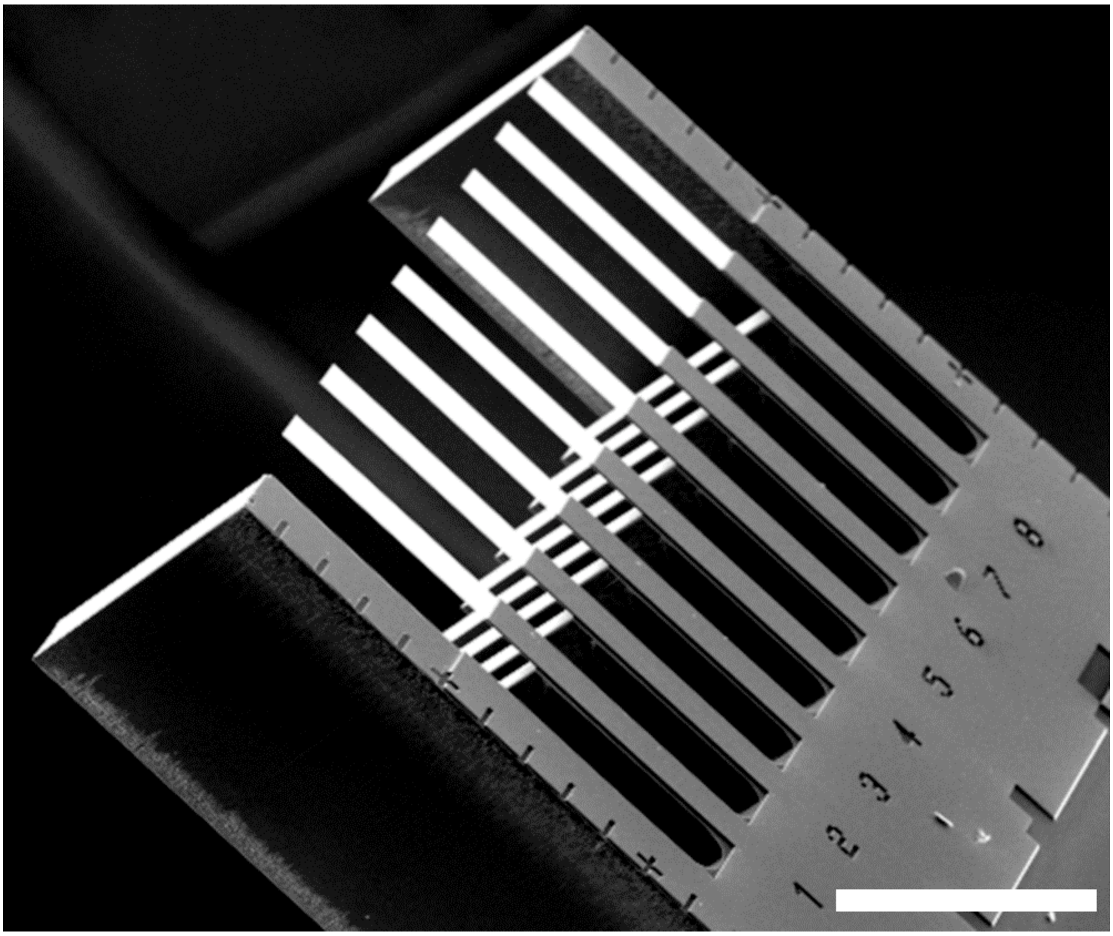

500µm

Supplementary Figure S1 *shows an array of eight cantilevers (thin bright bars) used for the measurement (scanning electron microscope (SEM) image). The dimension of an individual cantilever is: 500µm length, 100µm width and a thickness of 1µm. Note the protection bars at both sides.*
