# Supplementary material for: Comprehensive Characterization of Molecular Interactions Based on Nanomechanics
Source: PLoS One. 2008 Nov 3;3(11):e3610. doi: 10.1371/journal.pone.0003610 (PMC2572191; doi:10.1371/journal.pone.0003610)
Supplement: Figure S2 — (0.84 MB DOC) [file pone.0003610.s002.doc]

## Supplementary Figure S2


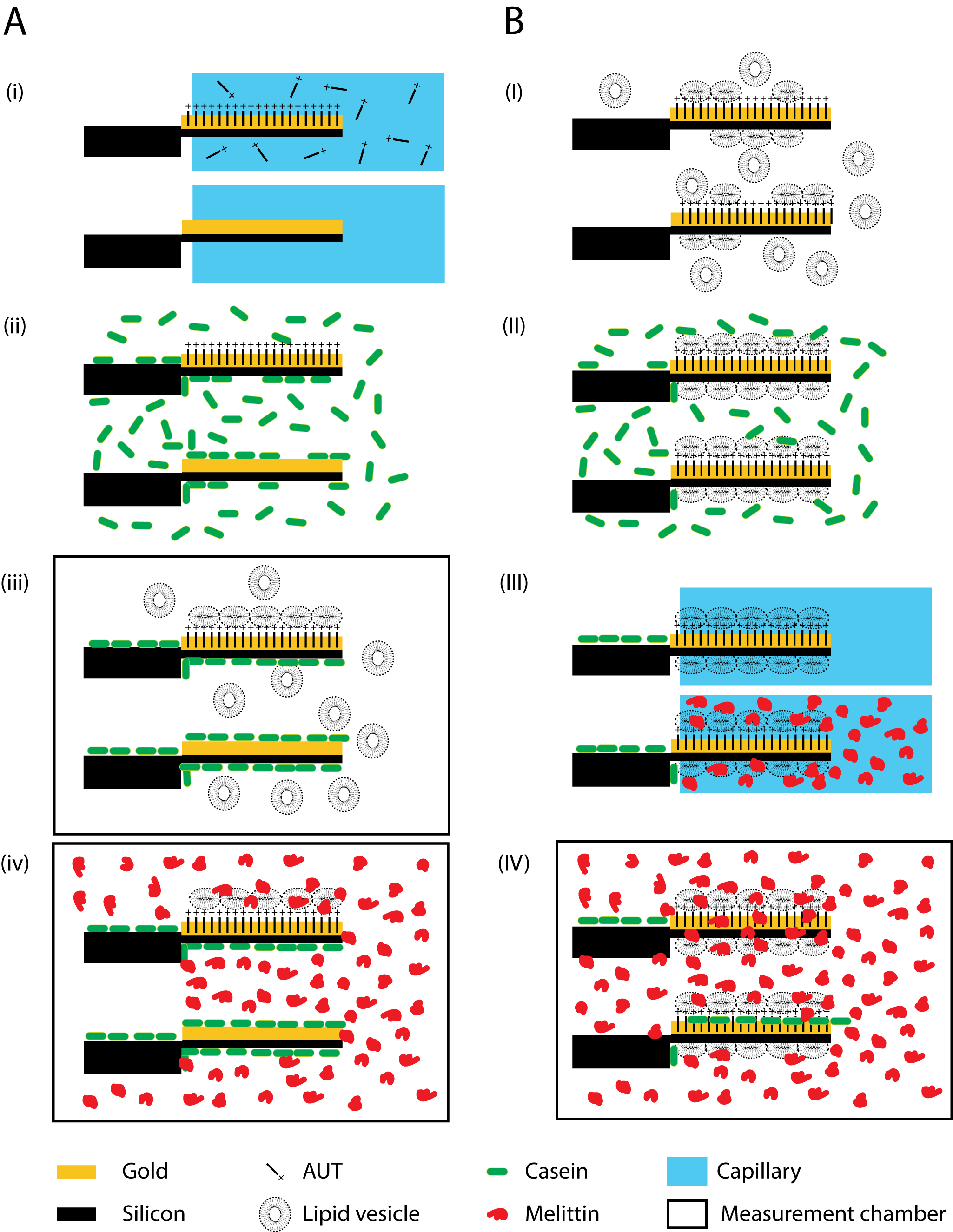


***Supplementary Figure S2*** *depicts the workflow of performed experiments presented in this work. Figure does not scale. The upper cantilever represents the positive control, the lower the negative control on the same cantilever array.*

**A** Experiment of manuscript:

(i) For the positive control, a self-assembled monolayer (SAM) of AUT was constituted using a capillary filled with a AUT solution. (ii) Afterwards, the whole array was incubated in a 1mg/ml casein solution for 10 min, efficiently blocking lipid and melittin binding (see **supplementary data S1**). (iii) The pre-functionalized cantilever array was mounted in the measurement chamber. After an initial buffer injection, lipid vesicles were injected. These vesicles specifically bind to the AUT functionalized cantilevers. (iv) After vesicle adsorption, again buffer was injected followed by a 1µM melittin solution.

**B** Experiment of **supplementary data S3**:

(I) After a SAM formation on the gold-coated cantilevers, the complete array was incubated in 0.5mg/ml lipid vesicles. Note that the lipid vesicles can bind at the AUT surface as well at the silicon surface. The asymmetry between the upper and lower cantilever surface important for cantilever bending is due to the AUT layer. This leads to different geometric arrangement of the lipid molecules. (II) Unspecific binding was blocked with a casein bath (1mg/ml, 20min). (III) The individual cantilevers were incubated in an array of micro-capillaries. By this way, every second cantilever was incubated in a 1 µM solution of melittin (negative control) whereas the other capillaries were filled with buffer. (IV) The prefunctionalized cantilever was mounted in the measurement chamber without drying and the melittin experiment was performed as shown in **the supplementary data S3**.
