# Supplementary material for: Comprehensive Characterization of Molecular Interactions Based on Nanomechanics
Source: PLoS One. 2008 Nov 3;3(11):e3610. doi: 10.1371/journal.pone.0003610 (PMC2572191; doi:10.1371/journal.pone.0003610)
Supplement: Data S1 — (0.33 MB PDF) [file pone.0003610.s003.pdf]

Supplementary data S1

This supplementary data presented here document the specific asymmetric functionalization of the cantilevers used for the experiments presented in the manuscript.

Figure S1-1 presents the mass adsorption experiments of cantilevers functionalized as follows:

- **Casein:** One side was gold coated, afterwards the cantilever was incubated 10 min in a 1 mg/ml casein solution
- **Si:** Cleaned Si cantilever (with oxidized surface) without further treatment
- **AUT one side:** One side of the cantilever was gold coated, and a SAM layer of AUT was constituted on the gold surface. Afterwards the cantilever was incubated for 30min in 1mg/ml casein solution.
- **AUT both side:** Both sides of the cantilever were coated with gold. Otherwise the cantilever was prepared exactly the same way as AUT/Gold-Si cantilevers.

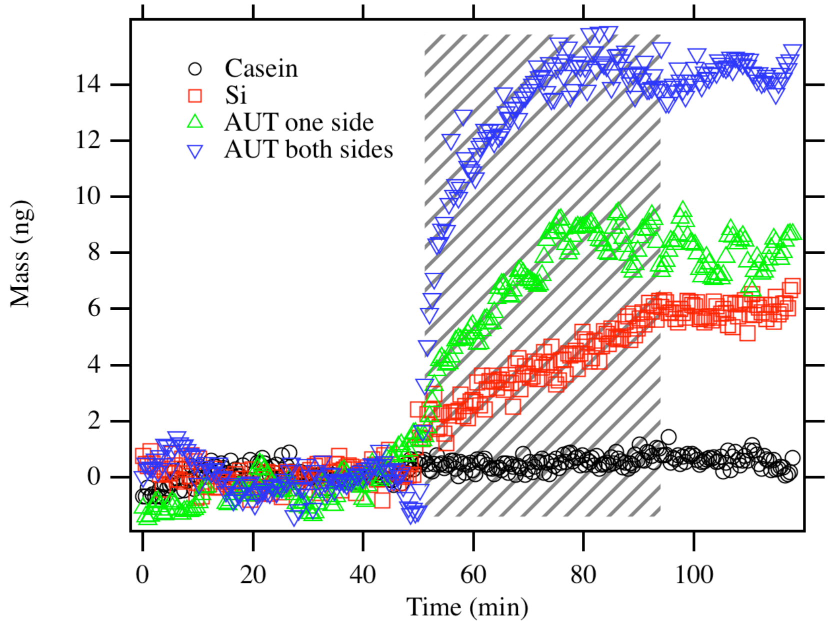

Figure S1-1*: Vesicle adsorption experiment of different functionalized cantilevers. The parameters for the vesicle injection are identical to the conditions in the manuscript.*

These results show clearly, that cantilevers with AUT on both sides of the cantilevers bind roughly the double amount of lipid than cantilevers only with one cantilever surface side pre-functionalized. Casein blocks efficiently the binding lipid vesicles on silicon cantilevers. Further more, silicon cantilever surfaces bind less lipid-vesicles (2.3 times less) than AUT pre-functionalized ones. Furthermore the adsorption kinetic is much slower and does not exhibit the clear saturation behavior as the AUT functionalized ones, even with a longer incubation times. The amount of bound lipid vesicles on AUT is only compatible with a layer of intact lipid vesicles and not with a one layer of broken up vesicles forming a supported bilayer. However, for untreated silicon cantilevers the conformation of the lipid is not that clear and we expect a mixture of broken and intact vesicles.

Table S1-1 documents that the chosen functionalization is specific for lipid adsorption and melittin does not bind:

Functionalization:	*Lipid-vesicles*	*Melittin*
Gold + AUT	+	-
Silicon + casein	-	-
Gold + Casein	-	-
Gold	-	-
Silicon	+	NA
Silicon + lipid	x	+
Gold + AUT + lipid	x	+

Table S1-1*: Performed control experiments to be in command of the specific functionalization for lipid vesicles and melittin (+: binding, -: no binding: x, Not applicable, NA: Not an answer).*
