# Supplementary material for: Comprehensive Characterization of Molecular Interactions Based on Nanomechanics
Source: PLoS One. 2008 Nov 3;3(11):e3610. doi: 10.1371/journal.pone.0003610 (PMC2572191; doi:10.1371/journal.pone.0003610)
Supplement: Data S2 — (0.37 MB PDF) [file pone.0003610.s004.pdf]

Supplementary data S2

Mass adsorption experiments with single sided, AUT functionalized cantilevers. Different concentrations of lipid-vesicles were continuously injected.

Figure S2-1 shows the mass adsorption versus time:

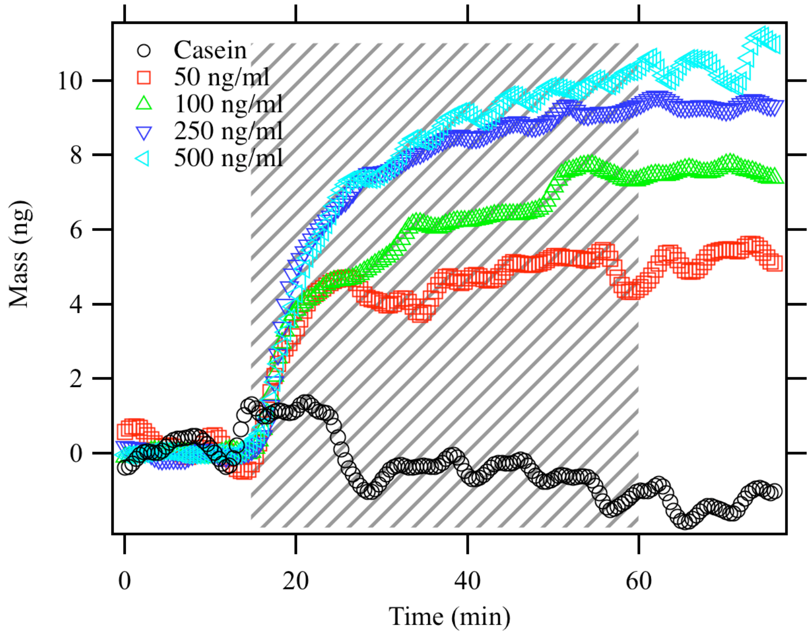

Figure S2-1*: Vesicle adsorption kinetic with differently concentrated lipid-solutions.*

Figure S2-2 shows the end-concentration of the adsorbed lipid-vesicles. The averages of 2 independent measurements (different cantilever arrays) are presented. Error bars represent standard errors.

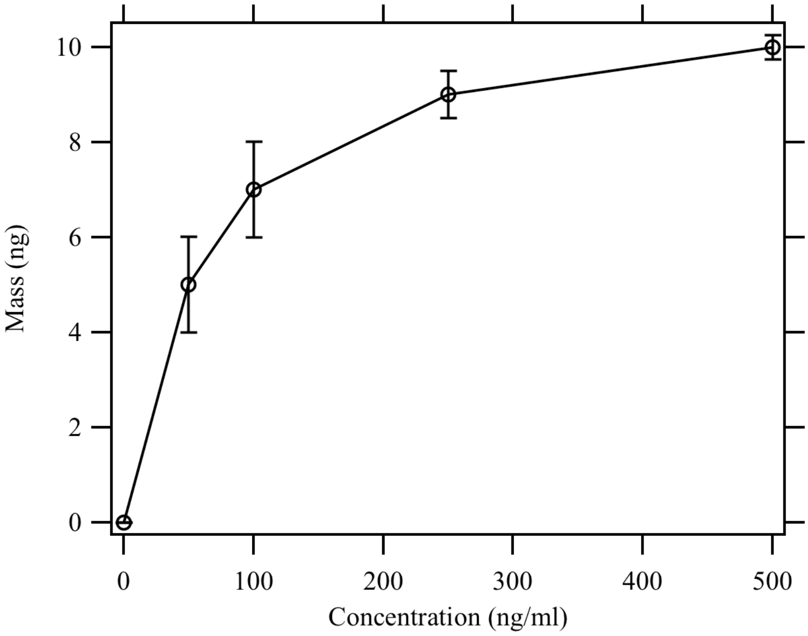

Figure S2-2*: End-masses form figure B1 versus lipid-vesicle concentration.*

Note that the vesicle solutions were flown continuously through the chamber and that the different end-masses are not due to depletion from lipid in the measurement chamber. We explain this

"saturation" like adsorption curve with the large flexibility of the vesicles and that the end-concentration is kinetically controlled: After the initial contact of the vesicle with the cantilever surfaces the vesicles are flattened. This flattening is limited by neighboring vesicles and with lower vesicle concentration an individual vesicle has more time to settle down and can occupy a larger area before getting in contact with neighbors. The saturation is due to the minimal area, which a vesicle occupies.
