# Supplementary material for: Comprehensive Characterization of Molecular Interactions Based on Nanomechanics
Source: PLoS One. 2008 Nov 3;3(11):e3610. doi: 10.1371/journal.pone.0003610 (PMC2572191; doi:10.1371/journal.pone.0003610)
Supplement: Data S3 — (0.20 MB DOC) [file pone.0003610.s005.doc]

## Supplementary Data S3

**Figure S3-1** shows the result of a differently designed experiment compared to the results discussed in the manuscript (see **supplementary Figure S2**). In this experiment, the cantilever was first functionalized with an AUT layer on gold, then incubated in a lipid-vesicle solution under heating for 120min at 50°C. In a last preparation step, every second cantilever was incubated in a capillary device with Melittin. Note, Melittin binds to both sides using this procedure (**Supplementary data S1**) and the main difference between the two sides of the cantilever are a) the amount and b) the structure of the lipid vesicles. Note, that the bending direction cannot be compared directly to **figure 2** **of the manuscript** (lipid as well as melittin on both sides of the cantilever).


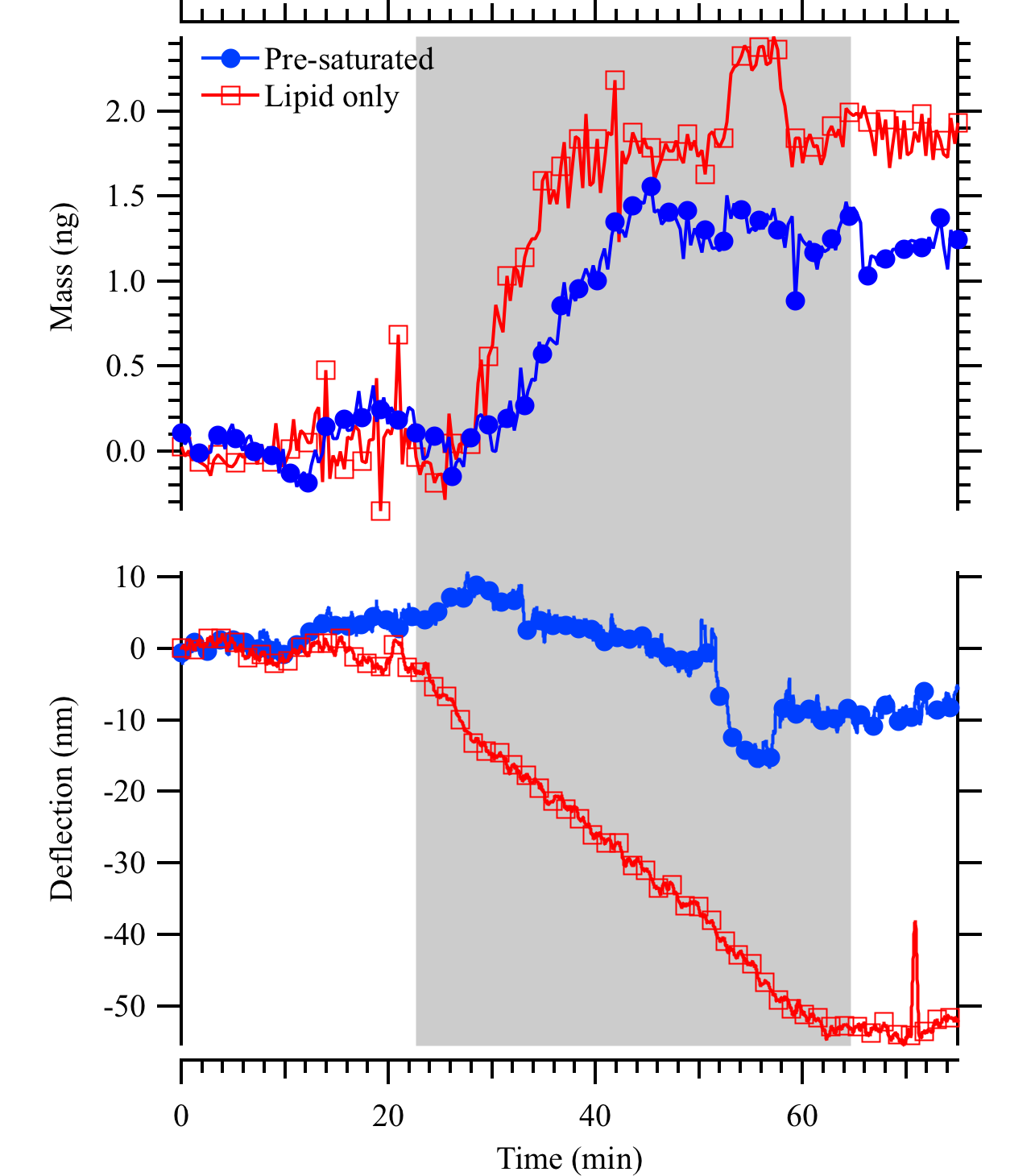


***Figure S3-1****: Melittin binding experiment on unsaturated lipid cantilevers (red open squares) and with melittin pre-saturated cantilevers (blue filled circles). Note that we show these data as raw data here since almost no drift is observed. In general, it is crucial to discuss the differential signal. The deflection direction cannot be compared to the results in the manuscript due to the different functionalization.*
