# Supplementary material for: Comprehensive Characterization of Molecular Interactions Based on Nanomechanics
Source: PLoS One. 2008 Nov 3;3(11):e3610. doi: 10.1371/journal.pone.0003610 (PMC2572191; doi:10.1371/journal.pone.0003610)
Supplement: Table S1 — (0.07 MB DOC) [file pone.0003610.s006.doc]

## Supplementary Tables S1

**Supplementary Table S1-A** Overview of the detected masses during the different buffer injections of Figure 2 in the manuscript. The data was extracted from the average of two independent cantilevers during the buffer injection when the signal stabilized (AVG: average, STDV: standard deviation, N: Number of measurement points from the average of two cantilevers, SE: standard error).

| *Section* | *AVG [ng]* | *STDV [ng]* | *N* | *SE [ng]* | *Rel. Error* |
| --- | --- | --- | --- | --- | --- |
| I | -0.2616 | 0.430 | 100 | 0.0430 | 16.41% |
| III | 6.087 | 0.372 | 79 | 0.0418 | 0.69% |
| V | 9.422 | 0.382 | 106 | 0.0371 | 0.39% |
| VII | 11.79 | 0.368 | 64 | 0.0460 | 0.39% |
| IX | 13.58 | 0.274 | 14 | 0.0732 | 0.54% |
| XI | 18.84 | 0.975 | 52 | 0.140 | 0.72% |

## Supplementary Table S1-B Summarizes the mass changes for the different injections for lipid and melittin of Figure 2 in the manuscript (the standard errors are estimated by the Gaussian error propagation):

| *Injection (sections)* | *AVG [ng]* | *SE [ng]* | *Rel. Error* |
| --- | --- | --- | --- |
| Lipid (III-I) | 6.348 | 0.060 | 0.95% |
| Melittin (V-III) | 3.335 | 0.056 | 1.68% |
| Lipid (VII-V) | 2.365 | 0.059 | 2.50% |
| Lipid (IX-VII) | 1.797 | 0.086 | 4.81% |
| Melittin (XI-IX) | 5.265 | 0.15 | 2.92% |

**Supplementary Table S1-C** Overview of the detected cantilever deflections during the different buffer injections of Figure 2 in the manuscript. Note that data were extracted from the differential signal between the positive functionalized and negative functionalized cantilevers (two independent cantilevers each).

| *Section* | *AVG [nm]* | *STDV [nm]* | *N* | *SE [nm]* | *Rel. Error* |
| --- | --- | --- | --- | --- | --- |
| I | -0.70 | 2.71 | 100 | 0.271 | 38.74% |
| III | 184.26 | 9.50 | 79 | 1.07 | 0.58% |
| V | 169.68 | 7.96 | 106 | 1.20 | 0.71% |
| VII | 331.88 | 5.12 | 64 | 1.20 | 0.36% |
| IX | 452.54 | 1.42 | 14 | 1.2 | 0.26% |
| XI | 347.99 | 1.47 | 52 | 1.20 | 0.34% |

## Supplementary Table S1-D Summarizes the deflection changes for the different injections for lipid and melittin of Figure 2 in the manuscript:

| *Injection (sections)* | *AVG [nm]* | *SE [nm]* | *Rel. Error* |
| --- | --- | --- | --- |
| Lipid (III-I) | 184.9 | 1.10 | 0.60% |
| Melittin (V-III) | -14.6 | 1.61 | 11.01% |
| Lipid (VII-V) | 161.9 | 1.69 | 1.04% |
| Lipid (IX-VII) | 120.6 | 1.69 | 1.40% |
| Melittin (XI-IX) | -104.5 | 1.69 | 1.62% |
